# Quantum dot-based thermometry uncovers decreased myosin efficiency in an experimental intensive care unit model

**DOI:** 10.3389/fphys.2024.1485249

**Published:** 2024-11-20

**Authors:** Meishan Li, Nicola Cacciani, Fernando Ribeiro, Yvette Hedström, Bhanu P. Jena, Lars Larsson

**Affiliations:** ^1^ Department of Clinical Neurophysiology, Karolinska Hospital, Stockholm, Sweden; ^2^ Department of Clinical Sciences, Comparative Medicine, Swedish University of Agricultural Sciences, Uppsala, Sweden; ^3^ Department of Anatomy, Institute of Biomedical Sciences, University of São Paulo, São Paulo, Brazil; ^4^ Department of Physiology, Wayne State University, Detroit, MI, United States; ^5^ Center for Muscle Biology, Viron Molecular Medicine Institute, Boston, MA, United States

**Keywords:** quantum dot, myosin, efficiency, ICU, critical illness myopathy

## Abstract

Critical illness myopathy (CIM) detrimentally affects muscle function in ICU patients, with a dramatic loss of muscle mass and function where the loss in specific force exceeds the loss in muscle mass (maximum force normalized to muscle cross-sectional area). The preferential loss of the molecular motor protein myosin, representing the hallmark of CIM, exhibiting a significant negative impact on the specific force generation by the muscle. Interestingly however, the preferential myosin loss is a relatively late event, and a specific loss in force generation capacity, is observed prior to the myosin loss. In the current study, employing an optimized cadmium telluride quantum dots (QD) mediated-thermometry approach to assess the efficiency of the myosin, we were able to determine the loss in specific force generated by the muscle, prior to the preferential loss of myosin. Reduction in QD fluorescent intensity correlates with greater heat loss, reflecting inefficient myosin function (less mechanical work performed and more heat loss on ATP hydrolysis by myosin). A significant decrease in myosin efficiency was observed in rats subjected to the ICU condition (immobilization and mechanical ventilation) for 5 days using an established experimental ICU model not limited by early mortality. Thus, qualitative myosin changes preceding quantitative myosin loss offer a mechanism underlying the early loss in specific force generation capacity associated with CIM and opens a venue for future CIM intervention strategies.

## 1 Introduction

Intensive Care Units (ICUs) have undergone significant development resulting in higher survival rates, due to improved medical technologies, progress in therapies, and the introduction of evidence-based medicine resulting in removal of harmful interventions. However, lifesaving ICU interventions are also associated with complications reflective of the acquired critical illness myopathy (CIM) with staggering negative consequences for patient quality of life, morbidity/mortality, and healthcare costs. CIM is observed in approximately 30% of ICU patients exposed to long-term mechanical ventilation and immobilization and 100% in some subpopulations (Friedrich et al., 2015; Cacciani et al., 2022a; Llano-Diez et al., 2012). ICU patients with CIM are characterized by flaccid paresis of limb muscles with craniofacial muscles being spared or less affected, massive loss of muscle mass and a preferential loss of the molecular motor protein myosin in limb muscles (for refs see (Friedrich et al., 2015)). However, the loss in muscle force is disproportionally larger than the loss in muscle mass resulting in a decreased specific force (maximum force normalized muscle cross-sectional area). The preferential myosin loss associated with CIM has a significant impact on the loss in specific force, but the loss in specific force precedes the preferential myosin loss in limb muscles (Cacciani et al., 2020). In the diaphragm, on the other hand, a dramatic loss in specific force is observed in the absence of a preferential myosin loss, but associated with myosin modifications (Corpeno et al., 2014).

In limb muscles, post-translational modifications (PTMs) of myosin preceding the preferential myosin loss in response to exposure to the ICU condition have been shown to play a significant role underlying in the early decline in specific force in an experimental ICU model (Cacciani et al., 2020), but it remains unknown how these myosin PTMs influence myosin function. The recent introduction of cadmium telluride quantum dots (CdTe QDs) mediated thermometry to measure myosin efficiency at the protein level has advanced our understanding of myosin function *in vitro* (Li et al., 2022; Laha et al., 2017; Kuhn et al., 2018). This approach is based on the detection of heat loss during ATP hydrolysis by myosin ATPase. i.e., the monitoring of temperature increases in microenvironment measured by QD fluorescence with a thermal resolution of ∼1 mK. This novel method is designed to indirectly measure the myosin ATPase hydrolysis reaction using a thermometric approach rather than directly measuring ATP hydrolysis products. Consequently, in this study, efficiency was defined through thermometric analysis rather than traditional enzymological measures (Li et al., 2022). Recently, we have optimized a method where myosin is extracted from muscle mini bundles with total protein being quantified to maintain the myosin-to-total protein ratio constant. Using this approach, myosin isoform differences in efficiency were demonstrated (Li et al., 2022), offering a novel approach using optimized QD-mediated thermometry, requiring minimal muscle tissue, to evaluate the impact of myosin PTMs induced by the ICU condition on the function of myosin.

In attempt to improve our understanding of the mechanisms underlying the impaired force generating capacity of limb muscles associated with CIM, we have used a unique experimental ICU model allowing long-term studies in rats developing CIM in response to controlled mechanical ventilation and immobilization, i.e., the ICU condition. QD-mediated thermometry was used to determine myosin efficiency prior to the preferential myosin loss.

## 2 Materials and methods

### 2.1 Animals and ICU model

Ten adult female Sprague-Dawley rats were divided into a sham-operated control group (CON, n = 5) and an experimental group (EXP, n = 5). The EXP group was exposed to deep sedation, post-synaptic neuromuscular blockade and controlled mechanical ventilation for 5 days. There was no significant difference in the body weights of the CON (303 ± 8 g) and EXP groups (311 ± 17 g). Similar soleus muscle weights were also observed in the CON (116 ± 20 mg) and EXP (112 ± 20 mg) groups.

All experimental animals were maintained in fluid and nutritional balance throughout the duration of the experimental procedures by introducing (a) intra-arterial solution (0.6 mL/h) containing 21 mL H_2_O, 24 mL 0.5 N lactated Ringer, 0.84 g oxacillin Na, 0.65 mg alpha-cobrotoxin, 0.3 mg vitamin K (Synkavite), 20 meq K^+^ (as KCl); and (b) an intra-venous solution (0.6 mL/h) containing 26 mL H_2_O, 16 mL 0.5 N lactated Ringer, 20% glucose (Baxter), 0.32 g oxacillin Na for the initial 24 h; then 8.5% Travasol amino acids (Baxter) and 20% Intralipid (Kabi) were added subsequently to provide adequate nutrients (Dworkin and Dworkin, 1990; Dworkin and Dworkin, 2004). Body temperature, peripheral perfusion and oxygen saturation (measured continuously with an infrared probe in a hindlimb paw, MouseSTAT, Kent Scientific corp.) were monitored and maintained in the physiological range. The sham-operated controls were anaesthetized with isoflurane, maintained in spontaneous breathing, received intra-venous and intra-arterial solutions, and killed within 2 h of the initial isoflurane anaesthesia and surgery.

During surgery or any possible irritating manipulation, the anaesthetic isoflurane level, that is, the minimum alveolar concentration (MAC) was >1.5%, which maintains the following states: (a) the electroencephalogram (EEG) was synchronized and dominated by high-voltage slow-wave activity; (b) mean arterial pressure, 90–100 mm Hg, heart rate maintained below 420 beats/min and (c) no evident EEG, blood pressure or heart rate responses to surgical manipulation. Isoflurane was delivered into the inspiratory gas stream by a precision mass-flow controller. After the initial surgery, isoflurane was gradually lowered (over 1–2 days) and maintained at MAC <0.5% during the remaining experimental period. Rats were ventilated through a coaxial tracheal cannula at 72 breaths/min with an inspiratory and expiratory ratio of 1:2 and a minute volume of 180–200 mL and gas concentrations of 40% O_2_, 56,5% N_2_ and 3% CO_2_, delivered by a precision (volume drift <1%/wk) volumetric respirator. Airway pressure was monitored continuously as well as end-tidal CO_2_ (EtCO_2_) and normocapnic condition maintained (EtCO_2_ = 37–45 mm Hg) as well as normoxia (SpO_2_ >90%). Intermittent hyperinflations (six per hour at 19–20 cm H_2_O) over a constant positive end-expiratory pressure (PEEP = 1.5 cm H_2_O) were set to prevent atelectases. Post-synaptic NMB was induced on the first day (150 µg α-cobrotoxin) and maintained by continuous infusion (187 µg/day). Mechanical ventilation started after the NMB induction avoiding hypercapnia and hypoxaemia. Experiments were terminated after 5 days. Female rats were preferred because of easier urine bladder catheterization for diuresis monitoring. The diuresis was maintained above 1 mL/h. In no case did animals show any signs of infections or septicemia. The ethical committees at Karolinska Institutet approved all aspects of this study.

### 2.2 Muscle tissues and bundle preparations

Slow-twitch skeletal muscles, i.e., the slow-twitch soleus (SOL) muscle was collected from the hindlimbs of young Sprague Dawley rats. The animal experiments were carried out according to the guidelines of the Swedish Board of Agriculture and approved by the ethical committees at Karolinska Institutet. The SOL muscle tissues were dissected into bundles of approximately 50 fibers (−5 mm long) in relaxing solution containing 50% (vol/vol) glycerol at 4°C and tied to glass capillaries, stretched to about 110% of their resting slack length. Afterwards the bundles were chemically skinned by treatment for 24 h at 4°C in a relaxing solution, and then stored at −20°C. Within 1 week after above skinning treatment, the bundles were cryo-protected by transferring them to relaxing solutions containing increasing concentrations of sucrose (0, 0.5, 1.0, 1.5, and 2.0 M) at 30-min intervals, and then frozen in liquid propane chilled by liquid nitrogen. The frozen bundles were stored at −140°C. Before the experiment, the bundle was incubated in sucrose solutions with decreasing concentrations (2.0, 1.5, 1.0 and 0.5 M) sequentially at 30-min intervals and then kept in the skinning solution at −20°C for 2 weeks or shorter prior to usage (Li and Larsson, 2010; Li et al., 2015a).

### 2.3 The extraction of myosin and the spectrophotometric quantification

A muscle mini bundle consisting of 10–20 fibers (that may be varied according to the measured concentration of extracted protein) was separated gently and incubated in a microtube with 20 μL high‐salt buffer (0.5 m KCI, 25 mm Hepes, 4 mm MgCl_2_, 4 mm EGTA, pH adjusted to 7.6 before adding 2 mm ATP and 1% β‐mercaptoethanol) at 4°C for 30 min. A part of the solution containing extracted myosin and other minor proteins was kept on ice for the spectrophotometric quantification of the concentration and QD-mediated thermometry, and the remaining solution was kept frozen for sequential 12% SDS-PAGE to determine the relative content of myosin among extracted proteins. The concentration of extracted proteins was quantified by conventional spectrophotometry using absorbance at 280 nm, i.e., protein A280 method (Nanodrop, Thermo Scientific), which does not require a standard curve, but the blank control (contains high-salt buffer only) and internal control (different concentration of BSA, such as 1, 2, 3 and 4 mg/mL) were applied for quality control. Every sample and control for the A280 method have been briefly and sufficiently vortexed and the quantification was repeated at least 3 times to ensure consistency. The extracted myosin was proportional to the extracted total protein. The A280 value of the extracted total protein was therefore used to represent the concentration of the extracted myosin.

### 2.4 The optimized QD-mediated thermometry of myosin enzymatic reaction

In this study, the saturated ATP concentration was determined as 5 mM. For the measurement of extracted myosin, the concentration was usually relatively high for a measurable reaction, then the preparation needs to be diluted to a series of concentrations from a relatively low to relatively high value, or *vice versa*. The lowest concentration with the fastest reaction velocity was determined as the optimal concentration. Then, 1 µL of extracted myosin was pipetted to a well containing 30 µL low‐salt buffer, followed by the addition of 1 µL QD (1 mg/mL) and 30 µL of the blank control (0 mM ATP) or ATP solutions (5 mM ATP), respectively. The addition of blank control or ATP solutions and the detection of fluorescence signal were performed at the same time to avoid inconsistencies during the time course. The QD fluorescence signal was recorded every 5 s for 2 min by a fluorescence spectrophotometer [TECAN, Infinite M Nano, Switzerland (Li et al., 2022)], while the excitation and emission wavelengths were fixed at 310 and 530 nm, respectively. The measurements for each preparation were performed in sextuplicate with both the blank control and ATP solution controlled at 25°C. The low salt buffer and QD were kept at room temperature (22°C) and the remaining solutions were kept on ice.

### 2.5 Myosin isoform expression and relative quantification

After myosin extraction, the mini bundle was placed in SDS sample buffer in a microfuge tube and stored at −80 °C. The composition of myosin heavy chain (MyHC) isoforms was determined by 6% SDS-PAGE. The acrylamide concentration was 4% (wt/vol) in the stacking gel and 6% in the running gel, and the gel matrix included 30% glycerol. Sample loads were kept small to improve the resolution of the MyHC bands (type I and IIa). Electrophoresis was performed at 120 V for 22 h with a Tris-glycine electrode buffer (pH 8.3) at 10 °C (SE 600 vertical slab gel unit; Hoefer Scientific Instruments, Holliston, MA, United States). The gels were silver-stained and subsequently scanned in a GS-900 Calibrated Densitometer (Bio-Rad). The volume integration function (Image Lab software 6.0, Bio-Rad) was used to quantify the relative amount of each MyHC isoform when more than one isoform was expressed. After the QD-mediated thermometry assay, the remaining myosin preparations were kept in urea buffer in a microfuge tube and stored at −80°C. The relative quantification of MyHC contents in total extracted protein was determined by 12% SDS-PAGE. After centrifugation and heating (90°C for 2 min) a volume of 4 µL was loaded on 12% SDS-PAGE. The total acrylamide and Bis concentrations were 4% (wt/vol) in the stacking gel and 12% in the running gel. The gel matrix included 10% glycerol. Electrophoresis was performed for 5 h with a Tris–glycine electrode buffer (pH 8.3) at 15°C (SE 600 vertical slab gel unit, Hoefer Scientific Instruments). The gels were stained with Coomassie blue (SimplyBlue SafeStain, Invitrogen), as this staining shows high reproducibility and the ability to penetrate the gel and stain all proteins present, i.e., allowing accurate quantitative protein analyses. The gels were subsequently scanned to determine the relative content of myosin heavy chains in relation to total extracted protein (Cacciani et al., 2020; Li and Larsson, 2010; Akkad et al., 2014).

### 2.6 Data analysis and statistics

QDs fluorescence signals were detected and normalized to the starting fluorescent value and the corresponding relative fluorescence intensity formed a negative hyperbolic regression plotted over time. The initial rate of the polynomial regression of the relative fluorescence intensity over time was calculated. The rate of the blank control was subtracted from the rate of the “real” reaction and then normalized to the protein concentration of the extracted myosin, indicating myosin efficiency. Each preparation was measured in sextuplicate, normalized and evaluated individually according to the criterion, i.e., calculated rates which fell outside one standard deviation were excluded and the remaining qualified rates were included, and rates with negative values (indicating null reactions) were excluded. Statistical analyses were performed by SigmaPlot software version14. The data were presented as mean ± standard deviation if not stated differently and analyzed by the Student’s unpaired t-test.

## 3 Results

### 3.1 Myosin isoform distribution and myosin efficiency

The soleus muscle is a slow-twitch postural muscle where the β/slow (type I) myosin heavy chain (MyHC) isoform is dominating. The relative amount of the type I MyHC isoform varied between 95% and 100% (97% ± 2%) in the muscle fiber bundles from the CON group and the remaining 0%–4% (3% ± 2%) being the fast type IIa MyHC isoform. In the EXP group, on the other hand, muscle fiber bundles from two rats expressed a higher content of the type IIa MyHC isoform (16% and 39%). We have previously shown a higher myosin efficiency in fast than in slow MyHC preparations using QD mediated thermometry (Li et al., 2022). The muscle fiber bundles expressing the higher type IIa MyHC isoform in the EXP group were therefore treated separately.

**FIGURE 1:**
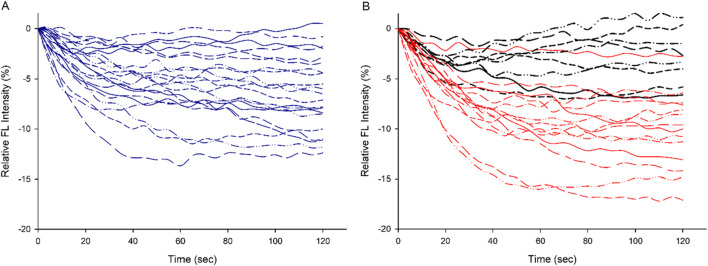
The individual plot of QD relative fluorescence intensity (the blank control subtracted from the experimental reaction) over time in CON [**(A)**, in blue] and EXP [**(B)**, in red. The black curves represent preparations with higher proportion of type IIa MyHC isoforms] groups, respectively, demonstrate lower myosin efficiency in the EXP group.

**FIGURE 2:**
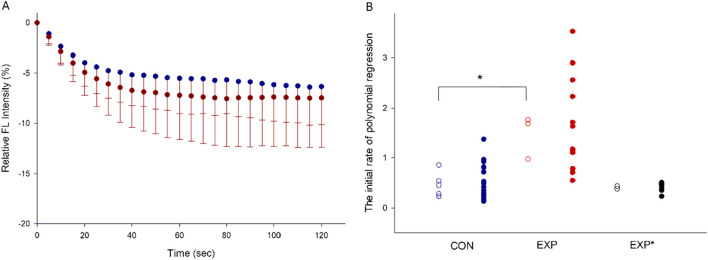
The averaged plot of relative fluorescence intensity over time. The values are presented as means–SD **(A)**. The distribution of the initial rate of polynomial regression are shown in the CON, EXP and EXP*(with higher proportion of type IIa MyHC) groups. The solid dots represent the qualified preparations, and the open circles represent the mean values per individual rat. (*P < 0.01, **(B)**.

QDs adhering to the myosin molecule are able to detect changes in the temperature of myosin during ATP hydrolysis and reflected by changes in fluorescence intensity. During the initial 0–20s of the QD thermometric reaction, the reproducibility of the data is high (Li et al., 2022). The following 20–120s measurements are noisy and are only used in the calculation of polynomial regression. The initial rate of polynomial regression after normalizing to the protein concentration of extracted myosin indicates myosin efficiency. The initial rate of polynomial regression for each rat is shown in CON and EXP rats expressing a high type I or IIa MyHC content (Figures 1A, B). A significantly higher (p< 0.01) myosin efficiency was observed in CON (0.47 ± 0.25) group than in the EXP (1.47 ± 0.44) group, i.e., a steeper decay of the QD fluorescence intensity in the EXP group (Figures 2A, B). The EXP* group with a higher type IIa MyHC content showed an initial rate of polynomial regression of 0.38 and 0.43, supporting an isoform related difference in myosin efficiency.

## 4 Discussion

Critical illness myopathy (CIM) is a common complication of modern critical care with significant negative consequences for patient quality of life, morbidity/mortality and healthcare costs. CIM is characterized by severe muscle weakness and atrophy where the weakness exceeds the loss in muscle mass. Interestingly, this early loss in force generating capacity is accompanied by decreased MyHC expression at the gene, but not at the protein level, thus preceding the preferential myosin loss in both clinical studies and in experimental ICU models (Cacciani et al., 2020; Ochala et al., 2011; Cacciani et al., 2022b; Ribeiro et al., 2024). However, the mechanisms underlying this initial loss in muscle function remain incompletely understood. Myosin is a mechanoenzyme that converts chemical energy from ATP hydrolysis into mechanical work, a process being isoform-dependent (Bottinelli et al., 1994; Weiss et al., 2001). However, our understanding of the properties of myosin at the muscle fiber level in the CIM model remains incomplete. In this study, we aimed to measure myosin efficiency to reflect the biochemical properties of myosin during the reaction. Quantum dots (QDs), semiconductor nanocrystals, were selected as nanoscale thermometers due to their superior fluorophores and thermal sensitivity compared with organic dyes or other fluorescent proteins (Laha et al., 2017; Kuhn et al., 2018). QDs have been successfully used to measure temperature changes during the ATPase reaction of purified myosin to determine myosin efficiency (Laha et al., 2017; Kuhn et al., 2018). A decrease in QD fluorescence intensity reflects a higher amount of heat production during ATP hydrolysis, indicating lower myosin work efficiency (Laha et al., 2017; Kuhn et al., 2018; Chern et al., 2019).

In animals exposed to the ICU conditions for 5 days, we observed a universal decrease in myosin efficiency in the SOL muscle in muscle bundles with a high type I MyHC content. The efficiency of fast myosin isoforms is higher than that of slow isoforms. In the two muscle fiber bundles with a higher content of fast (type IIa) MyHC isoforms was comparable to that of control muscle bundles with a high type I MyHC content indicating that myosin efficiency is universally decreased irrespective MyHC isoform expression suggesting a profound impact on myosin function in response to the ICU condition independent on myosin isoform.

Post-translational modifications of sarcomeric protein in response to mechanical ventilation and immobilization, i.e., the ICU condition, have been shown to contribute to an impaired muscle contractile function (Llano-Diez et al., 2012; Cacciani et al., 2020; Shanely et al., 2002). These chemical modifications alter functional properties through proteolytic cleavage or covalent attachment, removal, or exchange of a protein functional group. Thus, post-translational modifications are dynamic biological mechanisms regulating protein structure, localization, interactions and function (Morales et al., 2024). Mass spectrometry-based proteomic analyses of myosin obtained from respiratory and limb muscles from patients and animals exposed to the ICU condition show PTMs in the S1 head and rod region of the myosin protein (Llano-Diez et al., 2012; Cacciani et al., 2020; Corpeno et al., 2014; Llano-Diez et al., 2016; Addinsall et al., 2024). Thus, post-translational myosin modifications are forwarded as an important molecular mechanism contributing to contractile dysfunction and muscle weakness in response to the ICU condition.

Our group has previously reported oxidation, acetylation and methylation of myosin isoforms in the soleus in response to 5 days exposure to the ICU condition (Cacciani et al., 2020). Oxidative stress-induced alterations in the myosin motor domain, including the catalytic domain, significantly decreases myosin function (Corpeno et al., 2014; Shanely et al., 2002; Persson et al., 2019). Acetylation changes of myosin has been linked to reduced ATP turnover time of relaxed myosin molecules disturbing the energy demand of skeletal muscle in some congenital myopathic conditions associated with permanent muscle weakness (Sonne et al., 2023). Patients with heart failure also show significantly reduced acetylation of residues located in the myosin rod region and predicted to impact stability of thick filament rod interactions and ultimately myosin head positioning (Landim-Vieira et al., 2022). The methylation of myosin observed in both clinical and experimental studies in response to the ICU condition (Llano-Diez et al., 2012; Cacciani et al., 2020) may contribute to impaired myosin efficiency and structural stability, similarly as previously reported ageing-induced myosin methylation (Li et al., 2015b). Aligned with the key role of myosin PTMs on the muscle dysfunction in critically ill patients, an effective intervention to mitigate the ventilation-induced diaphragmatic dysfunction, the chaperone co-inducer BGP-15 was found to prevent the oxidation and methylation of specific residues located on the rod region of myosin and restore myosin function in both limb and respiratory muscles (Cacciani et al., 2020; Salah et al., 2016). Yet, further investigations are needed to understand whether PTMs in distant structural regions such as the myosin rod region contribute to decreased myosin efficiency observed in response to the ICU condition. Collectively, these findings support myosin post-translational modifications as an important molecular mechanism underlying the decline of myosin efficiency, contributing to impaired muscle force-generation capacity that precedes the preferential myosin loss observed following long-term mechanical ventilation and immobilization.

## 5 Conclusion

In conclusion, Critical Illness Myopathy (CIM) poses a significant challenge in the management of critically ill patients, characterized by severe muscle weakness and atrophy, attributed to a substantial reduction in myosin content and impaired function. This study utilized QDs as nanoscale thermometers to investigate myosin’s biochemical properties, revealing a universal decrease in myosin efficiency in the soleus muscle of animals exposed to ICU conditions for 5 days. Despite variations in myosin isoform composition, similar mechanisms likely underlie these findings. A deeper understanding of the molecular mechanisms governing myosin enzymatic dysregulation is essential for developing targeted interventions aimed at preserving muscle integrity and function in critically ill patients.

## Data Availability

The raw data supporting the conclusions of this article will be made available by the authors, without undue reservation.
